# Maternal COVID-19 Vaccination and Its Potential Impact on Fetal and Neonatal Development

**DOI:** 10.3390/vaccines9111351

**Published:** 2021-11-18

**Authors:** Niel A. Karrow, Umesh K. Shandilya, Steven Pelech, Lauraine Wagter-Lesperance, Deanna McLeod, Byram Bridle, Bonnie A. Mallard

**Affiliations:** 1 Department of Animal Biosciences, University of Guelph, Guelph, ON N1G 2W1, Canada; ushand@uoguelph.ca; 2 Department of Medicine, Faculty of Medicine, University of British Columbia, Vancouver, BC V5Z 1M9, Canada; spelech@shaw.ca; 3 Department of Pathobiology, University of Guelph, Guelph, ON N1G 2W1, Canada; lwagterl@uoguelph.ca (L.W.-L.); bbridle@uoguelph.ca (B.B.); bmallard@ovc.uoguelph.ca (B.A.M.); 4 Kaleidoscope Strategic Inc., Toronto, ON M6R 1E7, Canada; deanna@kstrategic.com

**Keywords:** COVID-19, SARS-CoV-2, vaccines, fetal development, neonatal development

## Abstract

Vaccines have been developed under accelerated timelines to combat the COVID-19 pandemic caused by the SARS-CoV-2 coronavirus. Although they are considered the best approach for preventing mortality, when assessing the safety of these vaccines, pregnant women have not been included in clinical trials. Thus, vaccine safety for this demographic, as well as for the developing fetus and neonate, remains to be determined. A global effort has been underway to encourage pregnant women to get vaccinated despite the uncertain risk posed to them and their offspring. Given this, post-hoc data collection, potentially for years, will be required to determine the outcomes of COVID-19 and vaccination on the next generation. Most COVID-19 vaccine reactions include injection site erythema, pain, swelling, fatigue, headache, fever and lymphadenopathy, which may be sufficient to affect fetal/neonatal development. In this review, we have explored components of the first-generation viral vector and mRNA COVID-19 vaccines that are believed to contribute to adverse reactions and which may negatively impact fetal and neonatal development. We have followed this with a discussion of the potential for using an ovine model to explore the long-term outcomes of COVID-19 vaccination during the prenatal and neonatal periods.

## 1. Introduction

Vaccines are a key strategy for preventing and controlling endemic and emerging diseases of both humans and livestock. In the case of COVID-19, which is caused by the zoonotic SARS-CoV-2 coronavirus, vaccines have been designed and produced under accelerated timelines, in part due to programs such as Operation Warp Speed [1]. Never before have vaccines been developed and made it to Phase III clinical trials in such a short period of time. The viral vector “Sputnik V” vaccine was the first to be registered in August 2020. The Oxford/AstraZeneca viral vector vaccine was later approved for use in the UK vaccination program in December 2020, and in that same month, the Pfizer–BioNTech mRNA vaccine was issued “emergency use authorization” by the US Food and Drug Administration, and was approved for individuals of 16 years of age and older in May 2021.

As of 23 October 2021, four different COVID-19 vaccines have been approved for use in Canada: Oxford/AstraZeneca’s Vaxzevria (Cambridge, UK), Pfizer–BioNTech’s Comirnaty (New York, NY, USA), Moderna’s Spikevax (MA, USA), and Johnson & Johnson’s Janssen (New Brunswick, NJ, USA) [1]. At this time, approximately 77% of the Canadian population that was 12 years or older had received at least one dose, and 84% was fully vaccinated, with most receiving the Pfizer–BioNTech and Moderna vaccines. Approximately 10% had received combinations of different vaccines, despite warnings against this “dangerous trend” by the World Health Organization (WHO), due to a lack of immunogenicity and safety data [2].

In terms of females, approximately 79% of Canadian females had received at least one dose and 75% were fully vaccinated at this point in time. Females mount a stronger immune response to vaccination than males, which can also make them more susceptible to adverse vaccine reactions (AVR) [3]. The number of COVID-19 vaccinated pregnant women in Canada is currently unknown, but it can be approximated using the numbers of births in Canada from 2001–2020 [4]. If we estimate 370,000 births per year, one can conservatively approximate that 243,581 pregnant Canadian women received at least one COVID-19 vaccine dose and that 231,248 were fully vaccinated within the 10-month vaccine rollout period based on the percentages of vaccinated females above. There is a concerted global effort underway to encourage pregnant women to get vaccinated despite the lack of safety data for this demographic [5], the rationale being that pregnant women who get COVID-19 are more likely to get critically ill and have adverse fetal/neonatal outcomes [6]. However, this rationale is not supported by all studies [7], and vaccination without long-term safety data from a randomized clinical trial and close medical oversight does not follow a precautionary principle, which is the standard of care in this group.

In the USA, 133,000 participants of the V-safe COVID-19 Vaccine Pregnancy Registry have indicated that they were pregnant at the time of vaccination, and the US Centers for Disease Control and Prevention (CDC) is currently enrolling eligible participants and analyzing data (i.e., pregnancy outcomes such as miscarriage and stillbirth, pregnancy complications such as preeclampsia and gestational diabetes, and problems with newborns such as preterm delivery, poor growth or birth defects) to better understand how COVID-19 vaccination may affect pregnant women [8]. A widely cited preliminary study of the V-safe and Vaccine Adverse Event Reporting System (VAERS) data [9] suggested that COVID-19 mRNA vaccines were safe for pregnant women [10]; however, an error was found in their analysis that undermined the original conclusion of safety in the context of spontaneous abortions [11], forcing a correction by these authors [12]. While these post-hoc data analyses of extreme clinical outcomes are important for assessing vaccine safety during pregnancy, they do not include more subtle multi-organ developmental changes that would be expected to occur in the fetus during an AVR, and these could lead to an increased risk of disease according to the Developmental Origins of Health and Disease (DOHaD) Hypothesis [13].

We have actually been advised to “feel positive about feeling bad” after receiving a COVID-19 vaccine [14]. However, the desired goal of these vaccines, to drive an anti-viral cell-mediated immune response against SARS-CoV-2 (i.e., the pro-inflammatory cytokines tumor necrosis factor (TNF) α, interleukin (IL)-1, IL-6, and Type I and II interferons (IFNs)), can also lead to adverse fetal outcomes [15,16].

## 2. Lipid Nanoparticles (LNPs) in the COVID-19 mRNA Vaccines

COVID-19 vaccine development has truly been unprecedented. Not only have vaccines been rapidly produced and approved for use, but this is the first time a coronavirus vaccine has ever been attempted for use on humans. Moreover, vaccines against infectious pathogens have not previously been created using the novel technologies that were used to develop the current emergency use COVID-19 vaccines. The mRNA vaccine platforms (Pfizer–BioNTech and Moderna) contain a genetically modified mRNA sequence encoding the immunogenic SARS-CoV-2 spike protein, which is used by the virus to invade host cells. They also contain a novel lipid nanoparticle (LNP) carrier system that allows for efficient endocytosis of the mRNA cargo by host cells. These LNPs possess adjuvant-like properties, both inflammatory and mRNA stabilizing, which is why conventional adjuvants are not required for these vaccines [17]. Nevertheless, the RNAs are not very stable when stored below −80 °C, and rapidly degrade at body temperature. The LNPs are comprised of ionizable cationic lipids, phospholipids, cholesterol and polyethylene glycols (PEGs), which are used to control the LNP size (60–100 nm), prolong circulation time and prevent LNP aggregation during vaccine storage [18]. Concerns were raised years ago regarding the safety of LNPs due to their biodistribution. For example, they were found to disperse to the ovaries in experimental mice [19]. Pfizer’s own pharmacokinetic studies of a surrogate vaccine containing ALC0315 and ALC0159 LNPs demonstrated that they dispersed over a 48 hours period to many rat endocrine and immune organs including the ovaries, adrenals, bone marrow, liver and spleen [20].

Very little is known about how LNP particle components are metabolized by the human body. Thus, further research must be completed, or information on studies from the companies that manufacture LNP components must be made available on Safety Data Sheets to indicate how these LNPs degrade into smaller catabolites. Research must also be conducted into how LNP components and their catabolites are distributed, retained and excreted. A critical component of the LNPs in both mRNA vaccines is the pegylated lipid, composed of a PEG unit with an average molecular mass of 2000 Da—DMG2000 in the Moderna and ALC-0159 in the Pfizer–BioNTech vaccine. Interestingly, smaller PEG molecules have been studied as a possible means for both inducing retinopathy and as a means for drug delivery to the eye. C57BL/6 mice were administered an intra-ocular injection of PEG8 to induce choroid neovascularization (CNV) after complement activation [21], and may serve as a model for studying macular degeneration of the retina. Dutch belted rabbits injected with PEG400 were reported to have retinal degeneration and atrophy 5 days post injection [22]. Both of these studies demonstrate that small sized PEGs can be toxic and, though helpful as a model for disease, indicate that pegylated lipids are an unsuitable method for intraocular drug delivery. As a follow up to this, two recent case reports published in the USA reveal a possible association with mRNA vaccines and damage to the retina. Subramony et al. [23], for example, reported a case of bi-lateral retinal detachment in a healthy 22-year-old after vaccination with the Moderna mRNA vaccine. This individual had no health issues, but upon ophthalmologic exam, was determined to have lattice degeneration. Post-vitreous retinal detachment is common in >50-year-olds due to the liquefied vitreous pulling away from the retina, but not in younger patients. Lattice degeneration by itself in this individual is unlikely to cause a retinal detachment; thus some other mechanism must have caused the retina to detach around this area of lattice. Fowler et al. [24] reported acute onset central serous retinopathy in a 33-year-old healthy male post-Pfizer—BioNTech vaccination. Given the known factors that cause acute central serous retinopathy, the author speculated on a few possible mechanisms as to why this occurred, including increased serum cortisol and free extracellular RNA—which can cause increased permeability of choroid endothelial cells—but also suggested that PEG may be involved, and mentioned the fact that PEG8 has been shown in mice to induce central serous retinopathy via the complement pathway. Without evidence of how catabolites of PEG in COVID-19 vaccines circulate and are excreted, one could hypothesize that PEG2000 molecules are broken down into smaller sizes, which could permit them to enter into the vasculature of immune-privileged tissues such as the eye and cause pathology.

LNPs are bioactive and the possibility of immunotoxicity has been raised. The innate immune system, for example, is activated when phagocytic cells (i.e., dendritic cells, macrophages, Kupffer cells, monocytes, mast cells and granulocytes) come into contact with LNPs, which are recognized as danger signals by host cell toll-like receptors (TLRs). Ligation of LNPs to these TLRs triggers the induction and release of abnormally high levels of pro-inflammatory and anti-inflammatory cytokines, referred to as cytokine release syndrome. LNPs can also activate serum complement, resulting in complement activation-related pseudoallergy (CARPA), which can lead to anaphylactic shock [25]. A recent pre-print study demonstrated that, when LNPs were injected intradermally into mice, inflammatory, pro-apoptotic, necroptotic and IFN gene pathways were induced, and when these LNPs were administered intranasally, 80% of mice died within 24 h [26].

PEGs have been previously used in both cancer immunotherapies and to deliver cytotoxins throughout the body. They have also been used to dampen cytokine and complement activation triggered by LNPs, but an optimal concentration of PEG is required to both maximize LNP protection from the immune system and to ensure thatthe LNP cargo remains bioactive [25]. PEGs were thought to have inert characteristics; however, it is now widely appreciated that they possess potent immunogenic properties. Exposure to PEG can result in the production of anti-PEG immunoglobulin (Ig)M and IgG, which can activate the complement system and result in anaphylaxis [27]. Anaphylaxis is one AVR that is associated with the COVID-19 mRNA vaccines [28], and for the Pfizer–BioNTech vaccine, the risk is 1:100,000 [29]. Since PEG is commonly used in consumer products, a considerable number of people may have already been sensitized to PEG and may therefore have pre-existing anti-PEG antibodies prior to COVID-19 vaccination [25]. Following endocytosis of the LNPs, PEGs can also freely interact with IgE antibodies that are bound to Fc receptors on mast cells and granulocytes; this can lead to Fc cross-linking that immediately triggers cellular degranulation, also resulting in anaphylaxis [30]. While anaphylaxis during pregnancy is typically a rare event, a recent study has reported severe outcomes for infants from mothers with anaphylaxis [31], which should alert us to potential fetal/neonatal outcomes resulting from vaccine-induced maternal anaphylaxis.

## 3. Viral Vector COVID-19 Vaccines

The COVID-19 viral vector vaccines (Oxford/AstraZeneca, Janssen, and Sputnik V) rely on adenovirus DNA vectors as carriers for the genetic information coding for the SARS-CoV-2 spike protein. Following intramuscular injection, the adenovirus invades host cells via receptor-mediated endocytosis. Its DNA is carried to the nucleus, and the host cell machinery then transcribes and translates it into spike proteins. Since, typically, 30 or more mRNA copies can be transcribed from a single DNA copy of this gene, this allows for a more marked amplification of total spike proteins than can be produced in the RNA-based vaccines.

The most commonly described side effects following the Oxford/AstraZeneca vaccination are injection site erythema, pain, swelling, fatigue, headache, fever and lymphadenopathy. However, in March 2021, vaccine-induced prothrombotic immune thrombocytopenia (VIPIT), also referred to as thrombosis–thrombocytopenia syndrome (TTS) or vaccine-induced immune thrombotic thrombocytopenia (VITT), was first reported for the Oxford/AstraZeneca vaccine. This should not be surprising, since thrombocytopenia has been consistently reported as an outcome of administering adenovirus vectors [32]. In April 2021, similar reports started to appear for the Janssen vaccine. Females less than 60 years of age are at the greatest risk of VIPIT within 5–30 days post-vaccination, and the estimated risk is 1:25,000 and 1:500,000 for the Oxford/AstraZeneca and Janssen vaccines, respectively [33]. In light of this AVR, many countries temporarily halted the use of these vaccines, but they were later reinstated, because the risk of COVID-19 was deemed greater than the risk of VIPIT. A number of hypotheses have been proposed to explain the potential mechanisms of VIPIT, including antibodies acting against platelet factor 4 (PF4), interactions between the adenovirus and platelets, cross-reactivity of SARS-CoV-2 spike proteins with PF4 (i.e., molecular mimicry), interactions between spike proteins and platelets, and platelet expression of adenoviral proteins [34]. With regards to pregnancy, a case study of immune thrombocytopenia was reported in a woman with mild COVID-19, and her newborn daughter who was COVID-19 free also experienced a decrease in platelet count that was resolved within 3 weeks postpartum [35]. In another study, a young COVID-19 positive woman who delivered a stillbirth at 29 weeks into gestation was also diagnosed with thrombotic thrombocytopenic purpura [36]. Interestingly, new-onset immune thrombocytopenia post-mild COVID-19 has been reported during the pandemic [37], and also following Pfizer–BioNTech vaccination [38], which indicates that the etiology of this condition is more complex than can be explained by the adenoviral vectors alone.

Rare neurological manifestations, such as Guillain–Barre syndrome (GBS), have also been reported to be associated with the COVID-19 adenovirus vector vaccines [39,40,41,42,43]. GBS is an acute inflammatory, demyelinating polyneuropathy characterized by progressive muscle weakness that is often self-resolving. However, in severe cases where respiratory muscles are compromised, it can be life-threatening and patients will require assisted mechanical ventilation [44], which has been reported to increase the risk of premature birth [45]. GBS is most commonly triggered by molecular mimicry following a gastrointestinal or respiratory illness. Mohkhedkar et al. [46] recently provided evidence to support the involvement of molecular mimicry in COVID-19 by identifying autoantibodies in cerebral spinal fluid from a GBS-diagnosed patient with COVID-19. It has also been proposed that pro-inflammatory cytokines and hypoxia may also contribute to COVID-19 related neuronal damage [43].

In addition to concerns about the adenovirus vectors, the Oxford/AstraZeneca vaccine also contains polysorbate 80 (Tween 80), which helps to stabilize the vaccine. This synthetic non-ionic surfactant has been previously used in various drug formulations [47]. However, polysorbate 80 is cross-reactive with PEG, so anti-PEG antibodies may also trigger an IgE-mediated hypersensitivity reaction to polysorbate 80 [28,48].

Lastly, concerns have been raised about the potential fate of foreign DNA in human cells, sourced from either an adenovirus vector, or reverse-transcribed mRNA coding the spike protein. While this is theoretically possible based on gene therapies, the likelihood of SARS-CoV-2 mRNA being reverse-transcribed into DNA and then integrating within the host genome is equally plausible during COVID-19 [49]; thus, the benefits of the COVID-19 vaccines for now are thought to outweigh the potential risk of DNA integration [50].

## 4. Bioactivity of the SARS-CoV-2 Spike Protein

The main host target receptor for the SARS-CoV-2 spike protein is angiotensin-converting enzyme 2 (ACE2), which is involved in maintaining blood pressure and vascular remodeling, and is expressed on adipocytes [51], other cells at mucosal surfaces, and in the vasculature, heart, kidneys, pancreas and brain [52]. ACE2 is also expressed within placental tissues [53], and is involved in regulating fetal myocardial growth and lung and brain development [54]. A recent pre-print study showed that blocking ACE2 with an anti-ACE2 antibody reduced placental SARS-CoV-2 infection [55]. This is one of a number of studies that have demonstrated that the placenta is susceptible to SARS-CoV-2 [54,56], and may also be responsive to spike proteins, which have been identified at low concentrations in plasma from recipients of the Moderna vaccine [57].

**Figure 1:**
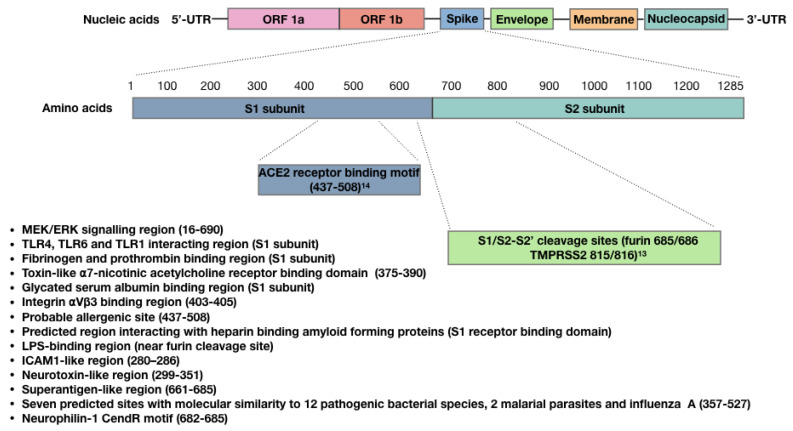
Sites within the SARS-CoV-2 spike protein S1 subunit and receptor binding domain, showing confirmed or predicted bioactivity.

A large number of studies have provided evidence that the SARS-CoV-2 spike protein is bioactive, and that ligation of the spike protein to ACE2 explains some of its bioactivity. A study by Lei et al. [58], for example, demonstrated that the spike protein down-regulated ACE2 in Syrian hamster vascular endothelial cells, which led to inhibited mitochondrial function and cell damage. In a later in vitro study, however, the spike protein was shown to upregulate bronchial epithelial cell ACE2 expression via activation of the Type I IFN signaling pathway [59]. These two studies indicate that the effect of this spike protein on ACE2 expression is tissue or species-specific. A recent study using mice and human umbilical cord blood demonstrated that ligation of recombinant spike protein to ACE2 can activate Nlrp3 inflammasome assembly, resulting in uncontrolled inflammation leading to pyroptotic cell death [60]. Ropa et al. [61] demonstrated that hematopoietic stem cells from human umbilical cord blood express ACE2 and were adversely affected by spike protein in terms of their ability to proliferate and expand into progenitor cells. Ropa et al. proposed that this could explain the reduced numbers of circulating lymphocytes and platelets that are observed in COVID-19 patients [61]. Using wild type and transgenic mice expressing human ACE2, Biancatelli et al. [62] recently demonstrated that intratracheal administration of the spike protein S1 subunit induced alveolar inflammation and acute lung injury and altered lung vascular permeability, leading to an ACE2-dependent systemic cytokine storm. Suzuki et al. recently demonstrated that the spike protein S1 subunit (Val-16-Gln-690), but not the ACE2 receptor binding domain (Arg-319-Phe-541), elicited mitogen-activated protein kinase (MEK/ERK) signaling in human pulmonary artery smooth muscle and endothelial cells [63]. These authors proposed that this growth factor/hormone-like cell signaling contributes to the hyperplasia and/or hypertrophy of vascular smooth muscle and endothelial cells in patients with COVID-19 and may also possibly explain some of the AVRs associated with COVID-19 vaccines [63]. By combining their knowledge of the SARS-CoV-2 spike protein, and the work of Chen et al. [64] on the SARS-CoV-1 spike protein conducted on human pneumocytes, Suzuki proposed that the spike protein functionally converts ACE2 from a peptidase to a functional cell membrane signaling receptor (Figure 1).

A number of studies have shown that the SARS-CoV-2 spike protein also possesses ACE2-independent bioactivity. Nader et al. [65] for example, found that in addition to ACE2, SARS-CoV-2 can also attach to, invade and damage host cells via αVβ3 integrin adhesion molecules, which are highly expressed on vascular endothelial cells. These authors demonstrated that an arginine–glycine–aspartic acid mutation (RGD motif) in this spike protein has uniquely allowed SARS-CoV-2 to acquire this function. Since the RGD motif is located adjacent to the ACE2 receptor-binding motif (Figure 1), this could allow SARS-CoV-2 to bind to cells lacking ACE2 and to potentially enhance binding to cells expressing both ACE2 and αVβ3 integrin. Interestingly, αVβ3 integrins are also expressed on platelets and contribute to platelet activation and aggregation [66]. Shen et al. showed that SARS-CoV-2 interacts with platelets to influence their function and promote dysregulated coagulation [67]; they proposed an ACE2-independent mechanism for this, because the expression of ACE2 is uncertain in platelets and their progenitor megakaryocytes [68]. It is possible that αVβ3 integrin is involved in SARS-CoV-2 interactions with platelets.

In silico analysis of the S1 subunit of the SARS-CoV-2 spike protein has revealed molecular docking sites for TLRs, including TLR1, TLR4 and TLR6; interactions with spike protein were the strongest for TLR4 [69] (Figure 1). An in vitro study performed by Shirato and Kizaki [70] demonstrated that the spike protein S1 subunit induced murine peritoneal macrophages to secrete pro-inflammatory cytokines via TLR4 signaling and that the response was attenuated using a TLR4 antagonist. TLR4 is also highly expressed in platelets, and when bacterial lipopolysaccharide (LPS) binds to TLR4, it can result in thrombocytopenia and the accumulation of platelets in the lungs [71]. Ouyang et al. [72] recently demonstrated that SARS-CoV-2 spike protein can also bind to bacterial LPS, and this spike protein-LPS interaction was shown to boost monocyte NF-κB activation and cytokine responses in vitro, as well as NF-κB activation in vivo [73]. Petruk et al. predicted the LPS interacting region to be within the proximity of the spike protein S1/S2 furin cleavage site (Figure 1), and proposed that spike protein–LPS interactions may in part explain the increased risk of severe COVID-19 caused by comorbidities.

The SARS-CoV-2 spike protein can also interact with other proteins. Grobbelaar et al. [74] demonstrated that when the spike protein S1 subunit was added to platelet-poor plasma, it interacted with and structurally modified plasma proteins β and γ fibrinogen, complement 3 and prothrombin, which made them more resistant to trypsinization. These authors proposed that this may contribute to the hypercoagulation associated with COVID-19 and may impair clot breakdown during fibrinolysis (Figure 1). The SARS-CoV-2 spike protein can also bind with high affinity to glycated human serum albumin. This may allow SARS-CoV-2 to evade the detection of its receptor-binding domain (RBD) by neutralizing antibodies (Figure 1); however, it can also lead to albumin depletion and may contribute to fluid tissue–vascular imbalance that can give rise to septic shock [75]. Also found within the RBD of SARS-CoV-2 and SARS-CoV-1 spike proteins is a “toxin-like” epitope that shares homology to snake venom α-bungarotoxin [76], which is a highly specific blocker of nicotinic acetylcholine receptors. Lagoumintzis et al. [77] hypothesize that the SARS-CoV-2 spike protein may block the cholinergic anti-inflammatory pathway, allowing for uncontrolled inflammation to occur during COVID-19.

The SARS-CoV-2 spike protein can also bind to the b1b2 domain of the neuropilin-1 receptor (NRP-1) [77], which normally interacts with vascular endothelial growth factor-A (VEGF-A) in neurons. ACE2 is not present in most neurons [78], although reports of neurological symptoms are common in COVID-19 patients [79]. Interestingly, interactions between the polybasic 682RRAR685 amino acid sequence, termed the “C-end rule” (CendR) motif (Figure 1), with NRP-1 potentiates SARS-CoV-2 entry into host cells [80]. This CendR motif is not conserved in either SARS-CoV-1 or Middle East respiratory syndrome coronavirus (MERS-CoV), and it is hypothesized that a “silencing” of pain through subversion of VEGF-A/NRP-1 signaling may underlie increased disease transmission in asymptomatic individuals [81].

There are three other regions of interest within the spike protein RBD that may also contribute to spike protein bioactivity. The first region is predicted with high probability to be an allergenic sequence [30]. This region could therefore contribute to anaphylaxis in some patients that have received viral vector and/or mRNA COVID-19 vaccines. The second RBD region of interest potentially allows the spike protein to bind to amyloid-forming heparin-binding proteins, which could lead to accelerated aggregation of amyloid proteins within the brain [82]. This supports Classen’s concern that COVID-19 vaccines could potentially induce prion disease [83]. The third region of interest within the RBD contains seven predicted molecular sites that share similarities to different toxins or virulence factors from 12 different bacterial species, 2 malarial parasites and influenza A [84] (Figure 1).

There is one final aspect of this spike protein that warrants consideration regarding its bioactivity, and this stems from the hypothesis that COVID-19-associated multi-system inflammatory syndrome in children (MIS-C) and the cytokine storm observed in adult patients with severe COVID-19 is mediated by spike protein superantigenic activity. Rivas et al. [85] have built on this hypothesis, first by drawing parallels between these two COVID-19 conditions and toxic shock syndrome (TSS). The superantigen *Staphylococcus* Enterotoxin B (SEB), associated with TSS, is a biotoxin that causes polyclonal T-cell activation and proliferation, which leads to massive production of pro-inflammatory cytokines. These researchers used structure-based computer modelling to discover an SEB-like sequence (glutamic acid661–arginine685) near the spike protein S1/S2 cleavage site that exhibits high binding affinity to both the T-cell receptor (TCR) β chain and co-stimulatory molecule CD28 [86] (Figure 1). They also identified several neurotoxin-like sequences within the spike protein; one (threonine299–tyrosine351) also displayed a high tendency to bind to the TCR, and another is an ICAM1-like region (aparagine280–threonine286) that is predicted to stabilize interactions between the spike protein and the TCR. These researchers also demonstrated TCRV/β skewing of the T cell response in COVID-19 patients with more severe and hyper-inflammatory clinical courses, which is consistent with spike protein superantigen activity. Additionally, they showed that the SARS-CoV-2 mutation aspartic acid839–tyrosine predictably enhanced binding affinity of the spike protein to the TCR, and later this group also provided evidence that a repurposed anti-SEB antibody could prevent SARS-CoV-2 infection in vitro [87].

Collectively, the diverse bioactivity of the SARS-CoV-2 spike protein makes this an ideal target for the immune system to neutralize the virus, and all the current COVID-19 vaccine platforms have focused on this spike protein because it is highly immunogenic [88]. However, we should also be cognizant of these bioactive properties when designing COVID-19 vaccines to ensure that only nontoxic immunogenic portions of the spike protein are expressed, and that their expression is both temporally and spatially limited and does not provide selection pressure driving viral mutation. The mRNA vaccines have been designed to allow a host cell to express the spike protein in its cell membrane [89], and the expression of the spike protein throughout the body is dependent on the biodistribution of LNPs—which primarily relocate to the spleen and liver, but have also been found in various other tissues [17,20]. We currently have no idea how long spike proteins are expressed by different host cells and in what tissues spike protein expression can occur because biodistribution studies on the spike protein have not been carried out to date [20]. The mRNA sequence has also been modified by manufacturers, with the addition of proline residues at positions 986 and 987, which could allow them to reside longer in the plasma membrane [17]. A recent pre-print by Patterson et al. [90] indicated that a subset of monocytes from COVID-19 patients contained SARS-CoV-2 S1 mRNA and proteins for as long as 15 months post-acute infection. This raises the possibility of the spike protein being expressed by maternal immune cells in colostrum and milk from COVID-19 positive mothers; thus, the biodistribution of spike mRNA and protein could be especially relevant during lactation. A recent study by Golan et al. [91] suggested that biodistribution of mRNA to milk during lactation is not a concern, as none was detected in milk from 6 mothers 4–48 h post-Pfizer–BioNTech and Moderna vaccination. However, a study demonstrated that following COVID-19 mRNA vaccination, exosomes expressing spike protein could be detected in plasma up to 4 months post-vaccination [92], which is concerning because we, and others [93,94], have shown that exosomes can be shed in bodily fluids such as colostrum and milk.

Lastly, Zhang et al. [49] have provided evidence that SARS-CoV-2 sequences can become integrated into human genomic DNA, and Seneff and Nigh speculated that retrotransposons in sperm and embryos could theoretically copy and paste SARS-CoV-2 cDNA into the fetal genome, resulting in the expression of spike protein that could render the neonatal immune system defenseless to mount an immune response to a subsequent SARS-CoV-2 infection, due to immune tolerance to viral proteins [17].

## 5. The SARS-CoV-2 Spike Protein Triggers Autoimmune Responses

Autoimmune diseases can be triggered by viral infections and some vaccines, and are more common to females [3]. There is mounting evidence to support the hypothesis that SARS-CoV-2 infection is a risk factor for autoimmune disease in predisposed individuals [95,96,97,98]. Autoimmune diseases manifest as hyper-stimulated immune responses against autoantigens, which are normally tolerated by the immune system. The proposed mechanisms of autoimmune response during SARS-CoV-2 infection have been previously discussed [98,99] and include molecular mimicry, bystander activation, epitope spreading, and polyclonal lymphocyte activation by SARS-CoV-2 superantigens. Molecular mimicry describes structural similarities between SARS-CoV-2 antigens and autoantigens that are recognized by immune cells (i.e., cytotoxic T cells) and immunoglobulins (i.e., autoantibodies and antiphospholipid antibodies) in cross-reactive epitopes. When autoantigens are targeted by these effectors, this can lead to immune-mediated tissue damage, and if autoreactive memory B-cells and T-cells are generated, this can lead to chronic disease. Bystander activation involves immune-mediated tissue damage resulting from a nonspecific and over-reactive antiviral innate immune response, such as the cytokine storm that has been described in severely impacted COVID-19 patients. In this case, tissue and cellular components become exposed during damage, and are then ingested by phagocytic cells and presented as autoantigens to autoreactive T helper and cytotoxic T cells, which contribute to ongoing immune-mediated pathology. Epitope spreading refers to ongoing sensitization to autoantigens as the disease progresses, which can lead to progressive and chronic disease. A recent study by Zuo et al. [100] implicated anti-NET antibodies as potential contributors of COVID-19 thromboinflammation; NETs are neutrophil extracellular traps that are produced by hyperactive neutrophils that have either come into contact with SARS-CoV-2 or have been activated by platelets and prothrombotic antibodies. These NETs are cytotoxic to pulmonary endothelial cells, and Zuo et al. discovered that anti-NET antibodies contribute to NET stabilization, which may impair their clearance and exacerbate thromboinflammation. Very recently, NETs were also implicated in VIPIT following the Oxford/AstraZeneca vaccine [101], but the potential involvement of anti-NET antibodies remains to be determined.

SARS-CoV-2 spike protein superantigen activity was discussed earlier. Superantigens are known to trigger the cytokine storm that can lead to immune-mediated multiple organ dysfunction syndrome, and this is often followed by immune suppression that can lead to persistent infection [102]. Superantigens such as SEB have been shown to exacerbate autoimmune disorders (i.e., experimental autoimmune encephalomyelitis and experimental multiple sclerosis) in mice models [103]. Recently, Jacobs proposed that long-COVID could be due in part to SARS-CoV-2 superantigen-mediated immune suppression, leading to persistent systemic SARS-CoV-2 infection [99]. In terms of pregnancy, prenatal exposure of rats to SEB was shown to attenuate the development and function of regulatory T cells in adult offspring [104] and alter the behaviour (i.e., increased anxiety and locomotion) of mice offspring [105].

Among the proposed mechanisms contributing to autoimmune responses during COVID-19, molecular mimicry has recently taken the front stage. A number of studies have found homologies between SARS-CoV-2 amino acid and human protein amino acid residues [106,107], and more specifically, between the spike protein and human proteins [108,109,110]. Additionally, some of these cross-reactive regions were immunogenic epitopes, meaning that they can bind to MHC I or II molecules on antigen-presenting cells, thereby activating autoreactive B and T cells that elicit an autoimmune response. Martínez et al. [109] for example, identified common host-like motifs in the SARS-CoV-1 and SARS-CoV-2 spike proteins nested in B and T cell epitopes. Morsy and Morsy also identified SARS-CoV-2 spike protein epitopes for MHC I and II molecules that were cross-reactive with the homeobox protein 2.1 (NKX2-1) and ATP-binding cassette sub-family A member 3 (ABCA3) lung proteins [110]. Kanduc and Shoenfeld searched for overlapping SARS-CoV-2 spike protein hexa- and hepta-peptides across mammalian proteomes and found a large number of matches within the human proteome; these authors stated that this is evidence of molecular mimicry, contributing to SARS-CoV-2-associated diseases [108]. Dotan et al. [111] also recently identified 41 immunogenic penta-peptides within the SARS-CoV-2 spike protein that are shared with 27 human proteins related to oogenesis, placentation and/or decidualization, implicating molecular mimicry as a potential contributor to female infertility. Vojdani and Kharrazian also demonstrated that anti-SARS-CoV-2 human IgG monoclonal antibodies cross-reacted with 28 out of 55 human tissue antigens derived from various tissues (i.e., mucosal and blood–brain barrier, thyroid, central nervous system, muscle and connective tissue), and BLAST searches revealed similarities and homologies between the SARS-CoV-2 spike protein and human proteins [112]. In terms of the COVID-19 vaccines, molecular mimicry has also been implicated in myocarditis, an AVR associated with the COVID-19 mRNA vaccines [113]. Huynh et al. [114] also recently identified autoantibodies as the potential cause of VIPIT; these autoantibodies were found to bind to PF4 and allowed for Fc receptor-mediated activation of platelets, which could initiate coagulation, leading to thrombocytopenia and thrombosis. These findings have raised concerns over the possibility that anti-SARS-CoV-2 spike protein antibodies may be responsible for VIPIT. Greinacher A et al. [115] investigated this hypothesis and found that SARS-CoV-2 spike protein and PF4 share at least one similar epitope. However, when they used purified anti-PF4 antibodies from patients with VIPIT, none of the anti-PF4 antibodies cross-reacted with SARS-CoV-2 spike protein. They therefore concluded that the vaccine-induced immune response against the SARS-CoV-2 spike protein was not the trigger causing VIPIT.

Others have implicated antiphospholipid antibodies in both COVID-19 and VIPIT-related thrombosis. APA are present in 1–5% of healthy people and are associated with the risk of autoimmune antiphospholipid syndrome (APS), which is the most common form of thrombophilia, and is more common in young women. APS during pregnancy is a risk factor for poor maternal and fetal outcomes such as pregnancy-induced hypertension, fetal loss, placental abruption, abortion, thrombosis, preterm delivery, pulmonary embolism, neonatal mortality, fetal growth restriction, premature infants and increased neonatal admission to intensive care units [116]. Antiphospholipid antibodies are a heterogeneous group of autoantibodies that recognize anionic phospholipids and protein–phospholipid aggregates, and are used as a diagnostic biomarker of APS. Bacterial, viral and fungal infections can elicit the production of antiphospholipid antibodies, and molecular mimicry is the proposed mechanism by which this occurs [117]. For example, human β2-glycoprotein I, which contains a highly immunogenic five-domain glycoprotein, displays homology to several microbial peptides [118]. Anti-β2-glycoprotein I antibodies have been detected in COVID-19 patients, and anti-β2-glycoprotein I antibodies are considered to be the most pathogenic antiphospholipid antibodies in APS [117]. β2-glycoprotein I is able to bind to endothelial cells and anti-cardiolipin antibodies, which can result in APA-induced endothelial cell damage [119]. Zussman et al. [119] recently demonstrated that antiphospholipid antibodies were able to bind to placental mitochondria, leading to ROS production. These authors proposed that APA binding to β2-glycoprotein I and cardiolipin in mitochondrial membranes contributes to oxidative stress and placental dysfunction. Antiphospholipid antibodies are also generated in response to vaccination, most commonly reported for influenza vaccines [120]. Martirosyan et al. reviewed cases of paediatric Henoch–Schonlein purpura and lupus associated with influenza vaccines and suggested that long-term effects such as thrombosis could be expected, since antiphospholipid antibodies remained elevated in some lupus patients for at least 6 months post-vaccination [120]. In the case of COVID-19 and COVID-19 vaccine-related AVRs, the role of antiphospholipid antibodies remains controversial, and more data are needed to establish potential cause–effect relationships [121].

Very recently, anti-idiotypic antibodies were also proposed as an autoimmune response following SARS-CoV-2 infection [122]. In this study, Arthur et al. detected ACE2 autoantibodies in convalescent plasma from previously infected patients, which were also correlated with anti-spike protein RBD antibody levels. Since patients with ACE2 autoantibodies also had less plasma ACE2 activity, these authors hypothesized that the ACE2 autoantibodies were anti-idiotypic antibodies that could interfere with ACE2 function and contribute to post-acute sequelae of SARC-CoV-2 infection (PASC, or “long-COVID”). We are unaware of ACE2 autoantibody levels being assessed following COVID-19 vaccination, so this warrants further investigation.

Collectively, the above autoimmune responses triggered by infection with SARS-CoV-2 or the COVID-19 vaccines suggest potential negative outcomes on fetal and neonatal development, and this should be explored in future studies. As with APS, cytokine storms and thromboinflammation are of concern—as is the potential for autoantibody responses that could target fetal/neonatal proteins.

## 6. The SARS-CoV-2 Spike Protein and Antibody-Dependent Enhancement

While antibodies have a number of important effector activities against SARS-CoV-2, including limiting viral attachment to epithelial cells and viral neutralization, non-neutralizing antibodies that enhance viral entry into host cells can sometimes also be generated; this immunological phenomenon is referred to as antibody-dependent enhancement (ADE). Since the early days of the COVID-19 pandemic, concerns have been raised about the possibility of ADE occurring, as it has been reported that both SARS-CoV-1 and MERS-CoV infect various animal models via ADE [123,124]. Ricke [124] proposed that SARS-CoV-2 may leverage Fc receptors for host cell invasion, and this may contribute to cytokine storms, leading to adult multi-system inflammatory syndrome, and also infant MIS-C—the latter presumably being mediated by passive transfer of maternal anti-SARS-CoV-2 antibodies that have become bound to Fc receptors on infant mast cells or macrophages [125]. While the potential risk of this type of ADE occurring in response to COVID-19 vaccines remains unknown, experience with SARS-CoV-1 spike protein vaccines demonstrates that it is indeed a possibility which warrants further investigation [124].

A second type of ADE involves non-neutralizing antibodies binding to and then eliciting conformational changes to viral proteins that can lead to enhanced viral adhesion to host cells [123]. Liu et al. [126] recently screened a panel of anti-SARS-CoV-2 spike protein monoclonal antibodies derived from COVID-19 patients and found that some of these antibodies that bind to the N-terminal domain of the spike protein induce open confirmation of the RBD, which enhances the binding capacity of the spike protein to ACE2 and the infectivity of SARS-CoV-2. Interestingly, these infection-enhancing antibodies have been identified in both uninfected and infected blood donors and have been detected at high levels in severe COVID-19 patients; their presence in uninfected people implies that these individuals may be at risk of severe COVID-19 if they later become infected with SARS-CoV-2 [126]. Another recent study has suggested that people may be at risk of infection by the SARS-CoV-2 Delta variant if they were vaccinated against the Wuhan strain spike sequence because the Delta variant is well-recognized by infection-enhancing antibodies targeting the N-terminal domain of the spike protein [127].

A number of murine studies have demonstrated that non-neutralizing maternal antibodies can increase the risk of neonatal disease. For example, pregnant mice infected with different strains of Dengue virus (DENV) display maternal anti-DENV IgG that is passively transferred during gestation and enhances the severity of offspring disease (i.e., hepatocyte vacuolation, vascular leakage, lymphopenia and thrombocytopenia) following infection with the heterotypic strain [128], and breast feeding has been shown to extend the window of ADE [129]. In terms of anti-SARS-CoV-2 antibodies, the passive transfer of anti-SARS-CoV-2 neutralizing antibodies has been detected in milk samples collected from women with COVID-19 [130]; however, non-neutralizing antibodies were not assessed. Anti-spike protein antibodies (IgG and IgA) have also been detected in milk samples from lactating mothers who were vaccinated with SARS-CoV-2 mRNA vaccines [131]; however, their neutralization/non-neutralization status was not assessed. Therefore, we have no data to determine whether or not passive transfer of ADE can occur during SARS-CoV-2 infection or COVID-19 vaccination, and so this warrants further investigation.

## 7. Using an Ovine Model to Study Long-Term Outcomes of Maternal COVID-19 Vaccination

Assessing long-term outcomes of vaccination in pregnant women and their offspring under the DOHaD paradigm is difficult. Humans have a long-life span and a generation interval of between 26–30 years. Differences in gender and genetics contribute to variations in immune responses, and environmental factors such as socioeconomic status, comorbidities, diet, exercise and vices act as interacting variables.

Large animal models such as sheep offer numerous advantages for DOHaD research. Sheep have a generation interval < 1.5 years, a long gestation period of 145 days, and their fetal size and rate of development is comparable to humans [132]. The ovine respiratory and cardiovascular systems are physiologically similar to humans, which make them an ideal model for studying respiratory diseases, neonatal lung development and xenotransplantation [133]. Pregnant sheep have been used to study connections between maternal allergies and offspring lung development and risk of allergies [134,135]. Sheep are also considered ideal models for studying neurodegenerative disorders such as Alzheimer’s, Parkinson’s and Huntington’s diseases [136], and due to their susceptibility to scrapie, are widely used to study prion diseases [137,138]. Sheep are also a well-recognized biomedical model for studying immunology [139], vaccine development and safety [140,141], and have been extensively used by our group and others to study the impact of maternal inflammation during pregnancy on offspring development [142]. We have shown, for example, that transient inflammation lasting approximately 6 h, elicited by an immune challenge with *Escherichia coli* LPS endotoxin at gestation day 135, is sufficient to alter male and female offspring immune responsiveness at 4.5 months of age [143], and neuroendocrine responsiveness at 5.5 months of age [144,145]. Using this endotoxin model, we have explored candidate protein and miRNA biomarkers for assessing the acute-phase response (APR) to immune challenge [146,147], and characterized variations in the APR at the population level [148], showing a moderately heritable phenotype [149].

As this pandemic continues to evolve, there are many unknowns with regards to COVID-19 and DOHaD. For example, what will be the outcomes of newborn babies born to mothers who have COVID-19 during pregnancy? [150]. Furthermore, what will be the outcome of offspring from mothers who received COVID-19 vaccines [151], and will this depend on gestational stage of exposure? With recent evidence of decreased vaccine efficacy against new SARS-CoV-2 variants [152], and immunity waning over time [153], addressing vaccine safety during pregnancy is becoming increasingly important, as efforts are already underway in some countries to administer more booster immunizations. Since epigenetic mechanisms are believed to contribute to the risk of DOHaD [154], a systems biology approach to addressing these questions is warranted, and we believe that the ovine model offers unique advantages for assessing the long-term impact of maternal COVID-19 vaccination on offspring health. Spike protein biodistribution studies can be performed during pregnancy and lactation by harvesting various tissues after euthanasia, and vaccine protocols can be mixed (i.e., primary immunization with Pfizer–BioNTech followed by booster immunization with Moderna) to assess the efficacy and safety of mixed vaccines. Offspring health can be assessed in terms of neonatal growth, gut development and neuroendocrine–immune system function. If phenotypic changes are observed, then mRNA, microRNA and circularRNA sequencing can be performed using different tissues to better understand any potential epigenetic changes brought on by maternal vaccination. Since there is evidence that the SARS-CoV-2 spike protein is capable of binding to ovine ACE2 [155], and that ovine respiratory organ cultures are susceptible to SARS-CoV-2 infection [156], this model species could also be used to investigate the potential impact of molecular mimicry and ADE on neonatal health.

## 8. Conclusions

In closing, we currently have no data to assess the outcome of maternal COVID-19 vaccination on offspring health, and this may take years to generate. We believe that the ovine model can be used to rapidly assess potential concerns about the administration of COVID-19 vaccines during pregnancy, and that the knowledge gained will help us to predict potential health outcomes in human offspring, which could lead to the development of treatments to help mitigate any potential adverse outcomes.
