# Correction: Karrow et al. Maternal COVID-19 Vaccination and Its Potential Impact on Fetal and Neonatal Development. *Vaccines* 2021, *9*, 1351

**DOI:** 10.3390/vaccines10111925

**Published:** 2022-11-14

**Authors:** Niel A. Karrow, Umesh K. Shandilya, Steven Pelech, Lauraine Wagter-Lesperance, Deanna McLeod, Byram Bridle, Bonnie A. Mallard

**Affiliations:** 1 Department of Animal Biosciences, University of Guelph, Guelph, ON N1G 2W1, Canada; 2 Department of Medicine, Faculty of Medicine, University of British Columbia, Vancouver, BC V5Z 1M9, Canada; 3 Department of Pathobiology, University of Guelph, Guelph, ON N1G 2W1, Canada; 4 Kaleidoscope Strategic Inc., Toronto, ON M6R 1E7, Canada

## 1. Change in Abstract

In the original publication [1], the sentence “Vaccines have been developed at “warp speed” to combat the COVID-19 pandemic caused by the SARS-CoV-2 coronavirus.” is changed into “Vaccines have been developed under accelerated timelines to combat the COVID-19 pandemic caused by the SARS-CoV-2 coronavirus.”

## 2. Change in Main Body Paragraph

1. In Section: **1. Introduction**

First paragraph: The sentence “In the case of COVID-19, which is caused by the zoonotic SARS-CoV-2 coronavirus, vaccines have been designed and produced at “warp speed” to combat the pandemic.” is modified into “**In the case of COVID-19, which is caused by the zoonotic SARS-CoV-2 coronavirus, vaccines have been designed and produced under accelerated timelines, in part due to programs such as Operation Warp Speed [1]**.” 

Third paragraph: The sentence “However, this rationale is not supported by all studies [7].” is changed into “**However, this rationale is not supported by all studies [7], and vaccination without long-term safety data from a randomized clinical trial and close medical oversight does not follow a precautionary principle, which is the standard of care in this group**.” 

Fourth paragraph: The sentences “A widely cited preliminary study of the V-safe and Vaccine Adverse Event Reporting System (VAERS) data suggested that COVID-19 mRNA vaccines were safe for pregnant women [9]; however, errors were found in their analysis [10], and a follow-up re-analysis of the data revealed a cumulative incidence of spontaneous abortion of 7–8 times higher than the original author’s calculations, which was statistically higher than the typical average for pregnancy loss during the equivalent time period [11]. While this post-hoc data analysis of extreme outcomes will be very important for assessing vaccine safety during pregnancy, it does not include more subtle multi-organ developmental changes that would be expected to occur in the fetus during an AVR, and these could lead to an increased risk of disease according to the Developmental Origins of Health and Disease (DOHaD) Hypothesis [12].” is changed into “**A widely cited preliminary study of the V-safe and Vaccine Adverse Event Reporting System (VAERS) data [9] suggested that COVID-19 mRNA vaccines were safe for pregnant women [10]; however, an error was found in their analysis that undermined the original conclusion of safety in the context of spontaneous abortions [11], forcing a correction by these authors [12]. While these post-hoc data analyses of extreme clinical outcomes are important for assessing vaccine safety during pregnancy, they do not include more subtle multi-organ developmental changes that would be expected to occur in the fetus during an AVR, and these could lead to an increased risk of disease according to the Developmental Origins of Health and Disease (DOHaD) Hypothesis [13]**.”

2. In Section: **2. Lipid Nanoparticles (LNPs) in the COVID-19 mRNA Vaccines**

First and last paragraph: Typographical error: “LPNs” is changed to “LNPs”.

## 3. References

The original references 11 and 12 have been removed. And, the following 3 new references have been added. Therefore, the numbering of all the citations has been changed.

11. Lancet Commission on COVID-19 Vaccines and Therapeutics Task Force Members. Operation Warp Speed: implications for global vaccine security. *Lancet Glob Health* **2021**, *9*, e1017–e1021.
12. Sun, H. On Preliminary Findings of mRNA Covid-19 Vaccine Safety in Pregnant Persons. *N. Engl. J. Med.* **2021**, *385*, 1535–1536.
13. Meaney-Delman, D.M.; Ellington, S.R.; Shimabukuro, T.T. On Preliminary Findings of mRNA Covid-19 Vaccine Safety in Pregnant Persons. Reply. *N. Engl. J. Med.* **2021**, *385*, 1536.

The authors state that the scientific conclusions are unaffected. This correction was approved by the Academic Editor. The original publication has also been updated.
